# Supplementary material for: Ultrastructure and Spectral Characteristics of the Compound Eye of Asiophrida xanthospilota (Baly, 1881) (Coleoptera, Chrysomelidae)
Source: Insects. 2024 Jul 13;15(7):532. doi: 10.3390/insects15070532 (PMC11277293; doi:10.3390/insects15070532)
Supplement: Supplementary file 1 [file insects-15-00532-s001.zip › insects-3050393-supplementary.pdf]

Table S1. Comparisons of rhabdom organization of ommatidia among representatives of Cucujiformia.

Species	Family/ subfamily	Rhabdom type	Relationship between PR and CR	Arrangement of PR	Arrangement of CR	Contribution to CR by R7 and R8	Orientation of microvilli in PR	Orientation of microvilli in PR	Polarization sensitivity	Reference
Tbanasimus formicarius	Cleridae	open	contacted	fused	fused	unequal	perpendicular	multiple	?	Wachmann (1977) [2]
Trichodes apiarius	Cleridae	open	isolated	fused	fused	unequal	multiple	multiple	-	Wachmann (1977) [2]
Clerus mutillarius	Cleridae	open	contacted distally, isolated basally	distally fused	fused	equal	parallel	multiple	-	Wachmann (1977) [2]
Neotriplax lewisi	Erotylidae	open	contacted	fused	vertically aligned	unequal	perpendicular	perpendicular	+	Mishra and Meyer-Rochow (2006) [32]
Donacia simplex	Donaciinae	open	isolated	fused	fused	equal	parallel	perpendicular	+	Wachmann (1977) [2]
Lema lichenis	Crioeerinae	open	isolated	fused	fused	equal	parallel	multiple	-	Wachmann (1977) [2]
Adoxus obscurus	Eumolpinae	open	isolated	fused	fused	equal	parallel	multiple	-	Wachmann (1977) [2]
Orsodacne cerasi	Orsodacninae	open	isolated	fused	fused	unequal	multiple	multiple	-	Wachmann (1977) [2]
Zeugopbora flavicollis	Megalopodinae	open	isolated	fused	fused	unequal	multiple	multiple	-	Wachmann (1977) [2]
Cassida vibex	Hispinae	open	isolated	fused	fused	equal	multiple	multiple	-	Wachmann (1977) [2]
Clytra laeviuscula	Clytrinae	open	isolated	fused	fused	equal	perpendicular	multiple	?	Wachmann (1977) [2]
Agelastica alni	Galerucinae	open	isolated	fused	fused	unequal	multiple	multiple	-	Wachmann (1977) [2]
Agasicles hygrophila	Galerucinae	open	isolated	fused	fused	unequal	multiple	multiple	-	Fan et al. (2023) [14]
Asiophrida xanthospilota	Galerucinae	open	isolated	fused	segmented	R8 absent	multiple	multiple	-	current study
Callosobruchus maculatus	Bruchinae	open	isolated	fused	fused	equal	multiple	multiple	-	Du et al. (2023) [13]
Melasoma aenea	Chrysomelinae	open	isolated	fused	fused	equal	parallel	perpendicular	+	Wachmann (1977) [2]
Chrysomela varians	Chrysomelinae	open	contacted	fused	fused	equal	parallel	multiple	-	Wachmann (1977) [2]
Phytodecta viminalis	Chrysomelinae	open	contacted	fused	fused	equal	parallel	multiple	-	Wachmann (1977) [2]
Timarcha tenebricosa	Chrysomelinae	open	contacted	fused	segmented	unequal	perpendicular	multiple	?	Wachmann (1977) [2]
Anaglyptus mysticus	Cerambycinae	open	isolated	fused	fused	equal	parallel	multiple	-	Wachmann (1977) [2], Wachmann (1979) [35]

Species	Family/ subfamily	Rhabdom type	Relationship between PR and CR	Arrangement of PR	Arrangement of CR	Contribution to CR by R7 and R8	Orientation of microvilli in PR	Orientation of microvilli in PR	Polarization sensitivity	Reference
Xylotrechus arvicola	Cerambycinae	open	isolated	fused	fused	equal	parallel	multiple	-	Wachmann (1979) [35]
Plagionotus arcuatus	Cerambycinae	open	isolated	isolated	fused	equal	parallel	multiple	-	Wachmann (1979) [35]
Demonax transilis	Cerambycinae	open	isolated	isolated	fused	equal	parallel	multiple		Gokan & Hosobuchi (1979) [34]
Stenopterus ater	Cerambycinae	open	isolated	significantly reduced	fused	unequal	multiple	multiple	-	Wachmann (1979) [35]
Anaglyptus mysticus	Cerambycinae	open	isolated	loosely fused	fused	equal	parallel	multiple	-	Wachmann (1979) [35]
Molorchus minor	Cerambycinae	open	contacted	fused	fused	unequal	parallel	multiple	-	Wachmann (1979) [35]
Paraclylus exculus	Cerambycinae	open	contacted	fused	fused	equal	parallel	multiple	-	Gokan & Hosobuchi (1979) [34]
Strangalia nigra	Lepturinae	open	isolated	isolated	fused	equal	parallel	multiple	-	Wachmann (1979) [35]
Grammoptera sp.	Lepturinae	open	isolated	isolated	fused	equal	parallel	multiple	-	Wachmann (1979) [35]
Strangalia sp.	Lepturinae	open	isolated	isolated	fused	equal	parallel	multiple	-	Wachmann (1979) [35]
Leptura ochraceofasciata	Lepturinae	open	isolated	isolated	fused	equal	parallel	multiple	-	Gokan & Hosobuchi (1979) [34]
Pidonia puziloi	Lepturinae	open	isolated	isolated	fused	equal	parallel	multiple	-	Gokan & Hosobuchi (1979) [34]
Stenocorus meridianus	Lepturinae	open	isolated	isolated	fused	unequal	multiple	multiple	-	Wachmann (1979) [35]
Tetrops praeusta	Lamiinae	/	/	completely absent	fused	equal	parallel	/	-	Wachmann (1979) [35]
Dorcatypus tristis	Lamiinae	open	contacted	fused	segmented	unequal	perpendicular	perpendicular	+	Wachmann (1979) [35]
Mesechthisfatus fujisanus	Lamiinae	open	contacted	fused	segmented	unequal	perpendicular	perpendicular	+	Gokan & Hosobuchi (1979) [34]
Agapanthia villosoviridescens	Lamiinae	open	contacted	fused	fused	equal	parallel	multiple	-	Wachmann (1979) [35]
Monochamus alternatus	Lamiinae	open	contacted	fused	fused	equal	perpendicular	perpendicular	+	Wen et al. (2020) [6]
Nupserha marginalis	Lamiinae	open	isolated	fused	fused	equal	parallel	multiple	-	Gokan & Hosobuchi (1979) [34]

Species	Family/ subfamily	Rhabdom type	Relationship between PR and CR	Arrangement of PR	Arrangement of CR	Contribution to CR by R7 and R8	Orientation of microvilli in PR	Orientation of microvilli in PR	Polarization sensitivity	Reference
Agapanthia cardui	Lamiinae	fused	fused	fused	fused	equal	parallel	multiple	-	Wachmann (1979) [35]
Ergates faber	Prioninae	open	contacted	fused	segmented	equal	parallel	perpendicular	+	Wachmann (1979) [35]
Distenia gracilis	Disteniinae	open	contacted	fused	fused	equal	parallel	multiple	-	Gokan & Hosobuchi (1979) [34]

Abbreviation: PR, peripheral rhabdomeres; CR, central rhabdomeres.
